# Fluorine-Incorporated Biogenic Hydroxyapatite Enhances Socket Bone Healing via Addressing Macrophage-Mediated Inflammatory Response

**DOI:** 10.3390/bioengineering12040396

**Published:** 2025-04-07

**Authors:** Chengwu Liu, Leyao Xu, Junming Feng, Bo Yang, Kaidi Chen, Yuanxiang Liu, Xiayi Wu, Shiyu Wu, Zhipeng Li, Shoucheng Chen, Zhuofan Chen

**Affiliations:** Hospital of Stomatology, Guanghua School of Stomatology, Sun Yat-sen University and Guangdong Provincial Key Laboratory of Stomatology, Guangdong Research Center for Dental and Cranial Rehabilitation and Material Engineering, Guangzhou 510055, China; liuchwu@mail2.sysu.edu.cn (C.L.);

**Keywords:** alveolar socket preservation, biogenic hydroxyapatite, fluorine, macrophage polarization, bone healing

## Abstract

Biological hydroxyapatite (BHA) has been extensively employed in alveolar socket preservation, yet its clinical application is often compromised by delayed bone healing triggered by macrophage-mediated pro-inflammatory responses. Building upon our previous work, in which we successfully incorporated fluorine into BHA to develop fluorinated biogenic hydroxyapatite (FBHA) with superior physicochemical and biological properties, this study systematically investigated the effects of fluorine doping on macrophage-mediated osteoimmunomodulation and socket bone healing. The synthesized FBHA was characterized using SEM, EDS, and fluoride ion release assays to confirm fluorine incorporation. In macrophage co-culture models, FBHA demonstrated significant advantages over BHA, effectively suppressing *iNOS* and *TNFα* gene expression, reducing NO release, and inhibiting phagocytic activity in M1 macrophages. RNAseq analysis revealed that the M1 phenotype suppression might be mediated through enhanced cellular antioxidant activity. Moreover, in macrophage-conditioned microenvironments, FBHA significantly upregulated osteogenic gene expression and ALP activity of pre-osteoblasts. In vivo experiments demonstrated FBHA’s superior performance in alveolar ridge preservation, especially in new bone formation and mineralization inside sockets. Fluorine doping significantly boosted socket bone healing via suppressing the inflammatory response of macrophages and enhancing osteogenic differentiation of pre-osteoblasts. These findings provide valuable insights into the development of next-generation biomaterials for alveolar socket preservation.

## 1. Introduction

Alveolar socket preservation has emerged as a critical strategy to counteract ridge bone resorption following tooth extraction [1,2,3,4]. This procedure typically involves the placement of bone substitutes into the alveolar socket to maintain the alveolar ridge contour [4]. Both clinical and preclinical studies have consistently validated its efficacy in preserving ridge contour and minimizing buccal bone resorption [2,3]. Among various bone substitutes, biogenic hydroxyapatite (BHA) is extensively utilized due to its structural similarity to human bone, low degradation rate, and abundant availability [3]. Biogenic hydroxyapatite (BHA) refers to hydroxyapatite directly derived from biological metabolic processes or biomineralization, with common sources, including bovine bone, porcine bone, coral, and so on [5]. Compared to synthetic hydroxyapatite, BHA materials retain bioactive components and natural microstructures, exhibiting superior biological performance. However, despite these advantages, BHA’s clinical application is often hindered by its propensity to induce prolonged inflammatory responses, leading to delayed bone healing [6]. Compared to autologous bone grafts or natural healing, the bone regeneration timeline for BHA can extend up to six months or longer [3], presenting significant limitations in socket preservation. Emerging evidence suggests that the early inflammatory response triggered by BHA implantation is a primary factor contributing to these delays [7], underscoring the need for advanced biomaterials with enhanced pro-healing properties.

Macrophages, as key orchestrators of the innate immune response, play a pivotal role in modulating inflammation and tissue regeneration following biomaterial implantation [8,9]. Their functional states are broadly classified into two polarization types: the pro-inflammatory M1 phenotype and the tissue-reparative M2 phenotype [10]. Successful biomaterial integration and tissue regeneration typically involve a dynamic transition from M1 to M2 macrophages, creating a favorable microenvironment for bone repair [11]. However, a prolonged M1-dominated response can impede bone tissue regeneration and compromise biomaterial performance [8,11]. Despite the growing recognition of macrophages’ role in osteoimmunomodulation, traditional studies have predominantly focused on the direct interactions between BHA and osteogenesis-related cells, overlooking the immunomodulatory effects mediated by macrophages. This gap highlights the necessity of developing biomaterials capable of regulating macrophage polarization to optimize the healing process [12].

Fluorine, a vital trace element in bone metabolism, has garnered attention for its potential in enhancing bone regeneration and biomineralization [13]. Ion doping, particularly with fluoride, has emerged as an effective strategy to augment the physicochemical and biological properties of hydroxyapatite [14]. Previous studies have demonstrated that fluoride ions, at specific concentrations, can modulate the osteoimmune environment by influencing macrophage polarization, thereby improving bone regeneration [15]. Building on this foundation, fluorinated biogenic hydroxyapatite (FBHA) was developed, exhibiting superior physicochemical properties and enhanced biological performance [16,17]. Preliminary findings revealed that FBHA can directly promote osteogenic differentiation in vitro and enhance bone regeneration in animal models [16,18], suggesting its promise in addressing the limitations of traditional BHA. However, its osteoimmunomodulatory effects, particularly in the context of socket preservation, remain unexplored.

This study aims to investigate the potential of fluorine doping to enhance the macrophage-mediated immunomodulatory and osteogenic properties of BHA. To achieve this, we characterized the physicochemical alterations in FBHA and established an in vitro co-culture model involving biomaterials, macrophages, and pre-osteoblasts to assess macrophage polarization and subsequent osteogenic differentiation. Furthermore, an in vivo canine tooth extraction socket preservation model was employed to evaluate the osteogenic efficacy of FBHA. By elucidating the role of fluorine incorporation in modulating the osteoimmune environment and accelerating bone healing, this study seeks to provide insights into the development of next-generation biomaterials for socket preservation and potential clinical applications.

## 2. Materials and Methods

### 2.1. Preparation and Characterization of FBHA and BHA

BHA and FBHA were prepared following a chemical and thermal procedure detailed in our previous research [14]. Briefly, porcine bones were boiled for 3 h to remove impurities and calcined at 800 °C for 2 h to obtain BHA. BHA blocks were soaked in 0.25 M NaF solution for 24 h, followed by calcination at 700 °C for 3 h to create FBHA. Both materials were milled into 0.25–1 mm granules and sterilized for experimental use. The morphology and elemental analysis of BHA and FBHA surfaces were conducted via a scanning electron microscope and the energy-dispersive X-ray spectroscopy (EDS) mode (SEM, Hitachi Regulus8100, Nasu, Japan), respectively. Crystal characteristics of BHA and FBHA were examined using an X-ray diffractometer (XRD, Rigaku SmartLab SE, Takasago, Japan). Stoichiometric HA pattern (JCPDS card #72-1243) was used as a reference.

Extracts were prepared in accordance with our previous study [16]. Sterilized samples were immersed in serum-free DMEM (Gibco, Waltham, MA, USA) at a concentration of 100 mg/mL. To eliminate bias, 4 g BHA and FBHA were weighed and mixed with 40 mL DMEM culture medium, separately, then incubated at 37 °C for 24 h. To facilitate uniform interaction between DMEM and materials, the centrifuge tubes were oriented horizontally during the 24-h incubation at 37 °C. Following incubation, the supernatant was collected after centrifugation and filtered through a 0.22 μm filter membrane (Merck Millipore, Billerica, MA, USA).

The concentration of fluoride ions in the extract was measured using a fluorine-selective electrode (PF-202-C; Leici, Shanghai, China) in conjunction with an ion analyzer (Origin Dual Star; Thermo Scientific, MA, USA). The extract used for measuring fluoride ion release was refreshed daily, and the amount of fluoride ions released was quantified each day for 7 days.

### 2.2. In Vitro: Macrophage Responses with BHA and FBHA Extract

#### 2.2.1. Cell Culture and Cell Proliferation

Macrophages (RAW 264.7) and pre-osteoblasts (MC3T3-E1) were sourced from the Cell Bank of Shanghai Institute of Biochemistry and Cell Biology, China. The complete medium (subsequently abbreviated as DMEM) was prepared as follows: DMEM (Gibco, Waltham, MA, USA) supplemented with 10%pre-inactivated fetal bovine serum (FBS, Nobimpex, Herbolzheim, Germany) and 1% penicillin/streptomycin (Gibco, USA). For the ensuing experiments, the BHA and FBHA extracts were fortified with 10% FBS and 1% penicillin/streptomycin as described above. Cells were cultured in an environment maintained at 37 °C with 5% CO_2_.

To assess the biocompatibility of FBHA, the proliferation of RAW264.7 macrophages was evaluated. Cells were seeded in a 96-well plate (4000 cells/well) and cultured with DMEM, BHA extract, or FBHA extract for 1, 2, and 3 days. Then, cells were rinsed twice with PBS and assessed using the cell counting kit-8 assay (CCK-8, Dojindo Lab, Kumamoto, Japan). The OD value was measured after incubating for 2 h in the dark at 450 nm wavelength.

#### 2.2.2. In Vitro Model of Macrophage with FBHA Extract

To investigate the modulatory effects of FBHA on macrophages, LPS (L2630, Merck Millipore, USA) was utilized to activate the macrophages. Macrophages were seeded in a 6-well plate (10^6^ per well) and cultured in medium with 100 ng/mL LPS (defined as M1 type) and without LPS (defined as M0 type) for 24 h [19]. Subsequently, the cells were rinsed with PBS thrice. Following this, these macrophages were co-cultured with 2 mL DMEM, BHA extract, or FBHA extract for 24 h, respectively. The groups with M0-type macrophages served as a control model.

#### 2.2.3. qRT-PCR of Macrophage Polarization and Inflammation

To investigate the impact of the extract on macrophages at the gene level, quantitative real-time polymerase chain reaction (qRT-PCR) was employed. After 24 h culturing with DMEM, BHA, or FBHA extract, total RNA was extracted utilizing an RNA Purification Kit (ESScience, Shanghai China). The quality and concentration of the extracted RNA were evaluated with a spectrophotometer (Nanodrop, Thermo Fisher Scientific, Waltham, MA, USA). Subsequently, cDNA was synthesized from 500 ng of RNA using a reverse transcription kit (Hifair^®^ III SuperMix, Yeasen, Shanghai, China), adhering to the manufacturer’s protocol. qRT-PCR was then carried out with the qPCR SYBR Mix (Hieff^®^ qPCR SYBR Green Master Mix No Rox, Yeasen, China) in a 10 µL reaction volume, using the LightCycler^®^ 96 instrument (Roche, Basel, Switzerland). The primer sequences utilized in qRT-PCR are detailed in Table S1.

#### 2.2.4. Nitric Oxide (NO) Synthesis and Phagocytic Function of Macrophages

After culturing for 24 h, the production of nitrate and nitrite anions in the medium supernatant was assessed using the Griess method. This was completed using the Total Nitric Oxide Assay Kit (S0023, Beyotime Biotechnology, Shanghai, China), according to the manufacturer’s protocol. The cultures were mixed with an equal amount of Griess reagent, and the OD values of each group were measured with an ELISA reader (wavelength 540 nm) within 15 min. The phagocytic function of macrophages was tested by using allophycocyanin (APC) fluorescence-labeled yeast glucan Zymosan. The medium was replaced with one containing 200 μg/mL APC-labeled yeast glucan Zymosan and co-culture for 30 min. A flow cytometer was used to detect the phagocytosis ratio of yeast glucan Zymosan by macrophages.

#### 2.2.5. RNA-Seq Analysis

To explore the initial gene expression alterations and potential mechanisms of FBHA-associated macrophage polarization, RNA sequencing (RNA-seq) analysis was performed. After 6 h culturing with BHA or FBHA extract, the macrophages were harvested, and total RNA was isolated as described above. RNA-seq analysis was carried out by BGI Genomics, China. Differentially expressed transcripts were identified using significance thresholds of *p* < 0.05 and a fold change of log2FC = 0.585. To gain insights into the biological functions and signaling pathways affected by these transcripts, the Gene Ontology (GO), Kyoto Encyclopedia of Genes and Genomes (KEGG) enrichment analysis, and Gene Set Enrichment Analysis (GSEA, v4.3.2) were performed, which provided information on gene functions and the involvement of specific pathways.

### 2.3. In Vitro: Effects of FBHA-Conditioned Macrophage Polarization on Osteogenesis

#### 2.3.1. Macrophage-Conditioned Medium Preparation

After a 24 h incubation of LPS-activated macrophages with DMEM, BHA extract, and FBHA extract, the medium supernatants were meticulously harvested and collected, respectively. These macrophage-conditioned mediums were subsequently subjected to centrifugation at 1500 rpm for 5 min to clarify the samples and then stored at −80 °C for subsequent experiments.

#### 2.3.2. qRT-PCR and ALP Staining

Two experimental models were set to assess the impact of macrophage-conditioned medium on pre-osteoblasts. For the control model, MC3T3-E1 cells were co-cultured directly with DMEM, BHA extract, and FBHA extract, respectively. For the test model, MC3T3-E1 cells were co-cultured with the collected macrophage-conditioned medium of the three groups (DMEM, BHA, and FBHA), respectively. In detail, MC3T3-E1 cells were seeded in a 6-well plate (10^6^ cells per well) and cultured for 12 h before the conditioned medium was replaced. After a 24 h co-culture period with conditioned medium, cells were collected for total RNA extraction. The primer sequences utilized in the qRT-PCR are detailed in Table S2. Likewise, MC3T3-E1 cells (10^5^ cells per well) were cultured in 24-well plates with co-stimulation medium consisting of osteogenic induction medium and macrophage-conditioned medium with different treatments at a ratio of 1:1. After 7 days of culturing, the MC3T3-E1 cells were stained using an Alkaline Phosphatase Color Development Kit (C3206, Beyotime, China). This staining method was utilized to track and evaluate the osteogenic differentiation of MC3T3-E1 cells throughout the experimental period.

### 2.4. In Vivo: Alveolar Socket Preservation in Canine

#### 2.4.1. Establishment and Surgical Procedures of Animal Model

Six male Beagle dogs, approximately 18 months old and 15 kg in weight, with fully erupted permanent dentition, were used for this experiment. The beagles were individually housed indoors and maintained on a standard pelletized dog food diet with ad libitum access to water. A 2-week acclimatization period preceded the commencement of the experiment. All experimental protocols were sanctioned by the Institutional Animal Care and Use Committee of Sun Yat-sen University (IACUC-DD-17-1205), and all procedures were conducted in strict accordance with the guidelines of the Animal Ethical and Welfare Committee, Sun Yat-sen University.

Preoperative sedation was induced via intramuscular injection of 0.05 mg/kg atropine, followed by the administration of 0.17 mg/kg acepromazine (Vetranquil 1%, Ceva Tiergesundheit, Düsseldorf, Germany) for further tranquilization. General anesthesia was subsequently achieved with an intravenous injection of 2% pentobarbital sodium (30 mg/kg, Sigma, Livonia, MI, USA). In addition, local oral anesthesia was provided by submucosal injection of 20 mg/kg primacaine adrenaline (Satelec, Mérignac, France) prior to surgical intervention.

Surgical procedures (Figure 5A): sulcus incisions were performed in the second maxillary premolar (PM2) area, and full-thickness buccal-palatal flaps were elevated. The bilateral PM2 were precisely hemi-sected using a fissure bur and were atraumatically extracted by an experienced surgeon. A total of 24 alveolar sockets were created and randomly assigned into three groups (*n* ≥ 6): (i) Control group as blank control with no graft, (ii) BHA group grafted with BHA, (iii) FBHA group grafted with FBHA. Each socket in the grafted groups was filled with the corresponding biomaterial up to the level of the marginal bony crest. The extraction site flaps were then carefully secured over the socket entrance using resorbable sutures (Vicryl^®^5.0, Ethicon Inc., Raritan, NJ, USA). Postoperative care included intramuscular penicillin G sodium (300,000 i.u., Pen-B^®^, Pfizer Inc., New York, NY, USA) and 0.2% chlorhexidine rinses, every 2 days for 10 days.

#### 2.4.2. Sample Acquisition and Analysis Procedure

Following a 3-month healing period, the dogs were euthanized using an overdose of 4% pentobarbital sodium (120 mg/kg, i.v., Sigma, USA). Block biopsies, including the socket sites and two adjacent teeth, were then dissected from the maxilla. These samples were fixed in 10% neutral-buffered formalin for 10 days before collection for micro-CT and histomorphometric analysis.

#### 2.4.3. Micro-CT Analysis

Micro-CT scans were performed using micro-CT equipment (μCT50, Scanco Medical, Brüttisellen, Switzerland). The scan settings included 360° rotation, aluminum filter, 70 kV, 200 μA, with a pixel resolution of 20 μm. The acquired data were processed and reconstructed using the μCT50’s system default software (Version 2.0). Three-dimensional visualizations were created with MeVisLab (Version 3.0.2, MeVis Medical Solutions AG, Bremen, Germany) for detailed evaluation.

The alveolar ridge contour was assessed using micro-CT measurements (Figure 6A). The vertical distance (VD) was used to evaluate the resorption of the buccal bone crest, while the contour height (CH) and horizontal width (HW) denoted the alveolar ridge contour. To be specific, VD between the pristine buccal and palatal crests and HW in the mid-sagittal planes of healed sockets were determined as follows: drawing a vertical line (CVL) at the socket’s center, and two horizontal lines perpendicular to CVL were delineated at the top of the buccal and palatal crests. Then, the distance between these horizontal lines was defined as VD, and the width of the horizontal line within the ridge contour through the palatal crest was defined as HW. The distance between the line connecting the buccal and lingual bone crests and the summit or trough of the alveolar ridge over the vector was defined as CH.

To evaluate bone morphometry differences within the sockets, three cuboid volumes of interest (VOIs), each 1.5 × 1.5 × 1.5 mm^3^, were selected from the apical, middle, and coronal areas of each socket’s center (Figure 6B). Bone morphometric analyses were conducted in each VOI, with parameters, including bone volume fraction (BV/TV), bone mineral density (BMD), trabecular number (Tb.N), trabecular thickness (Tb.Th), trabecular separation (Tb.Sp), and structural model index (SMI), based on *Parfitt*’s report [20]. BV/TV indicates the ratio of new bone volume to the VOI’s total volume. BMD reflects the mineralization density of new bone, which symbolizes the bone maturation status. Tb.N, Tb.Th, Tb.Sp, and SMI represent the mean density, thickness, separation, and the ratio of plates to rods structure of trabecular bone in the VOI. BV/TV and BMD analyses were performed in all VOIs, while trabecular bone analyses (Tb.N, Tb.Th, Tb.Sp, and SMI) were conducted in the apical and middle VOIs due to the cortical bone healing in the coronal socket.

#### 2.4.4. Histomorphometric Analysis

The block biopsies were decalcified in EDTA, dehydrated in gradient ethanol, and embedded in paraffin. The specimens were sectioned in the buccal-lingual plane, parallel to the long axis of the extraction site. Subsequently, the slides were stained with hematoxylin and eosin. The centrally located slides were scanned using a digital slide scanner (Aperio AT2, Leica Biosystems, Wetzlar, Germany), and a PC-based image analysis system (Image-Pro Plus, Media Cybernetics, Rockville, MD, USA) was used for histomorphometric analysis. In the photomicrograph, the total socket area (S) and the area occupied by newly formed bone (NB), residual materials (RM), and non-mineralized tissue (NMT) in the socket were measured. Separated areas of NB, RM, and NMT were selected manually, and the proportions (%NB, %RM, and %NMT) within the socket area were measured. Histomorphometric measurements were performed by one experienced examiner twice.

#### 2.4.5. Statistical Analysis

Statistical analyses were performed utilizing GraphPad Prism software (version 9.0). The Shapiro–Wilk’s test was employed to assess the normality of distribution. Accordingly, one-way ANOVA was applied for comparisons among groups, with post hoc analyses carried out using Bonferroni-corrected *t*-tests. These values were presented as means ± standard deviation (SD), and the level of statistical significance adopted was *p* < 0.05.

## 3. Results

### 3.1. Characterization and Biocompatibility of BHA and FBHA

**Figure 1:**
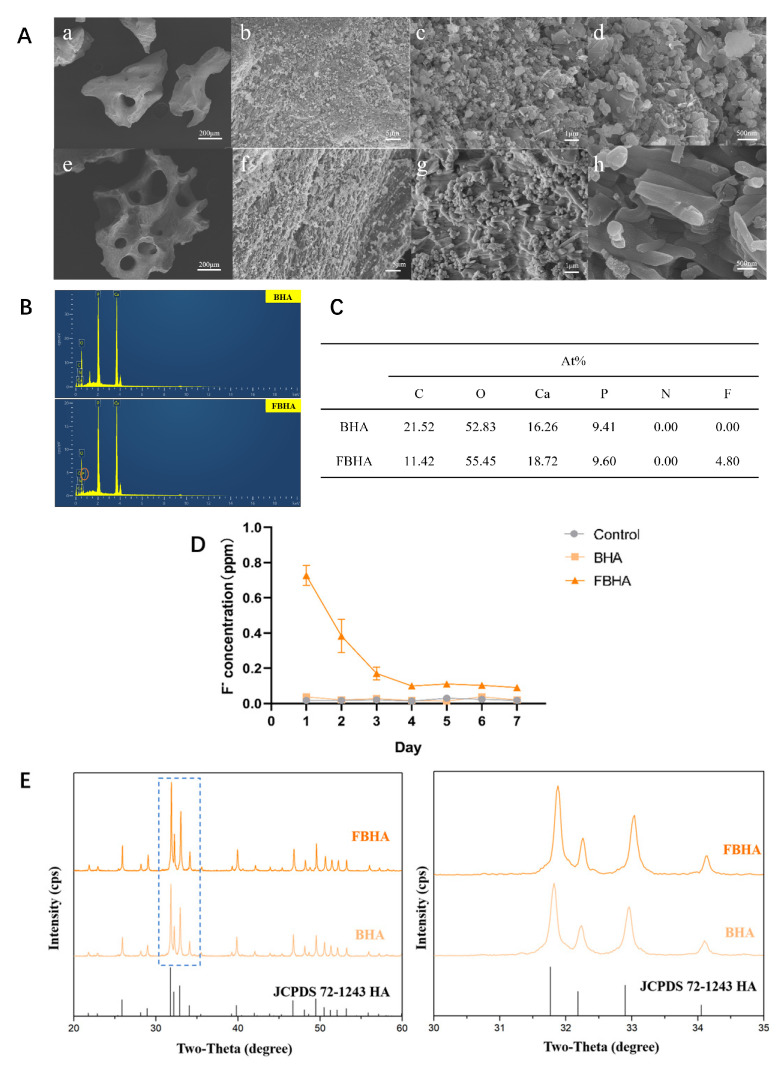
Characterization of fluorinated porcine hydroxyapatite. (**A**) SEM images displayed a spherical-shaped crystal of BHA (**a**–**d**) and a more rectangular crystal in FBHA (**e**–**h**), (**B**,**C**) EDS showed fluorine and other elemental distribution, (**D**) fluoride ion daily release showed a gradually reduced and sustained release of fluorine ion in FBHA, (**E**) XRD revealed a synchronous rightward shift of peaks in FBHA. Yellow circle in (**B**): Fluorine element; Blue dashed area of left figure in (**E**): Enlarged view shown at right.

BHA and FBHA were synthesized using a chemical and thermal method. SEM showed that both BHA and FBHA particles possessed natural porous structures with uniformly distributed crystals (Figure 1A). However, a distinct difference in crystal morphology was noted: BHA crystals exhibited a spherical shape (Figure 1A(d)), while FBHA led to a rod-like alteration in crystal form (Figure 1A(h)), indicating successful fluorine incorporation into the apatite lattice. In addition, EDS analysis confirmed the presence of fluorine elements in FBHA, which were absent in BHA (Figure 1B,C). Furthermore, the fluoride ion release curve showed a gradual release of fluoride ions, from 0.73 ± 0.06 ppm on day 1, followed by a reduced decrease to a stable level of 0.1 ± 0.02 ppm after day 4, and this concentration was sustained over time (Figure 1D). XRD revealed a synchronous shift in the peaks toward higher diffraction angles in FBHA, and the shifting was shown in the enlargement of the 30–35° (2θ) section (Figure 1E), which indicated HA lattice contraction, demonstrating the partial substitution of hydroxyl groups in HA with fluoride ions. These results suggested that fluoride was incorporated into the HA lattice through chemical substitution rather than physically adsorbed on the surface of BHA, which is consistent with our previous studies [17].

**Figure 2:**
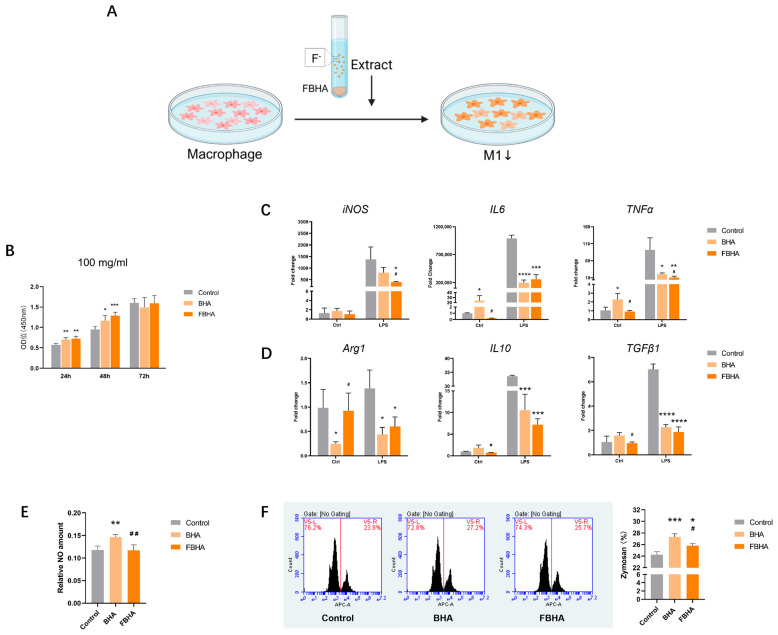
Biocompatibility and macrophage polarization of FBHA. (**A**) In vitro model of material extract culturing with macrophages; (**B**) Cck8 assay showed good biocompatibility in both FBHA and BHA. (**C**) Within the microenvironment of M1 macrophages, compared to BHA, gene expression of M1-related markers (iNOS, TNFα) was downregulated in FBHA, and (**D**) gene expression of M2-related markers was similar in FBHA and BHA. (**E**) NO release detection and (**F**) phagocytosis of Zymosan were downregulated in FBHA. *p* value: Compared with the Control group, *, *p* < 0.05; **, *p* < 0.01; ***, *p* < 0.001; ****, *p* < 0.0001. Compared with BHA group, #, *p* < 0.05; ##, *p* < 0.01.

CCK8 showed that both BHA and FBHA extracts enhanced the proliferative activity of macrophages on days 1 and 2, showing no significant cytotoxicity over the entire experiment (Figure 2B). Meanwhile, there was no significant difference between FBHA and BHA, indicating good biocompatibility of fluorine doping in BHA.

### 3.2. FBHA Extract Suppressed M1 Polarization

RT-qPCR analysis showed that, in both control groups, compared to non-activated macrophages, LPS-stimulated activated macrophages exhibited a significant upregulation in the expression of M1 polarization markers *iNOS* and pro-inflammatory factors *TNFα* and *IL6* (Figure 2C), while the expression of M2 polarization marker *Arg1* remained unchanged (Figure 2D). This indicated that LPS stimulation successfully induced the formation of an M1-polarized macrophage model.

Further, within the microenvironment of LPS-activated macrophages, also known as M1, significant differences in gene expression between FBHA and BHA were observed. Compared to BHA, FBHA significantly downregulated the gene expression of M1-related markers *iNOS* and *TNFα* (Figure 2C), indicating that fluorine doping could inhibit BHA-associated inflammatory response. At the same time, we found no significant difference between FBHA and BHA in the gene expression levels of M2 polarization markers *Arg1*, *TGFβ1*, and *IL10*, although both led to a decrease in gene expression compared to the Control group. This suggested that the FBHA extract might not have a direct promoting effect on M2 polarization in this FBHA extract.

In fact, in the microenvironment of non-activated macrophages, as known as M0, compared to the Control group, BHA significantly upregulated the gene expression of *IL6*, *TNFα*, and significantly downregulated the gene expression of *Arg1* (Figure 2D), which is consistent with previous studies, suggesting that bio-derived HA can cause a significant inflammatory response and promote M1 polarization [7]. Moreover, compared to BHA, FBHA significantly downregulated the gene expression of M1-related pro-inflammatory factors *IL6* and *TNFα* (Figure 2C), and promoted the upregulation of M2-related marker *Arg1* (Figure 2D). Overall, BHA may promote the expression of inflammatory factors, while fluorinated BHA can relatively suppress the BHA-associated inflammatory response.

Furthermore, the phagocytic activity of macrophages is considered to be related to the pathogen clearance function of M1 macrophages in the microenvironment [21]. We found that the Zymosan phagocytic activity of macrophages was significantly activated in the BHA group, while FBHA inhibited cellular phagocytic function compared to BHA (Figure 2F), which indicated that FBHA can relatively suppress the polarization function of M1 macrophages induced by BHA. On the other hand, in terms of NO release, which is closely related to the inflammatory function of M1 [22], FBHA significantly inhibited the increase in BHA-mediated NO release level (Figure 2E). These results exhibited that compared to BHA, FBHA could suppress the functional state of M1 macrophages.

### 3.3. RNA-Seq Indicated FBHA-Mediated Macrophage Polarization Through Antioxidation

**Figure 3:**
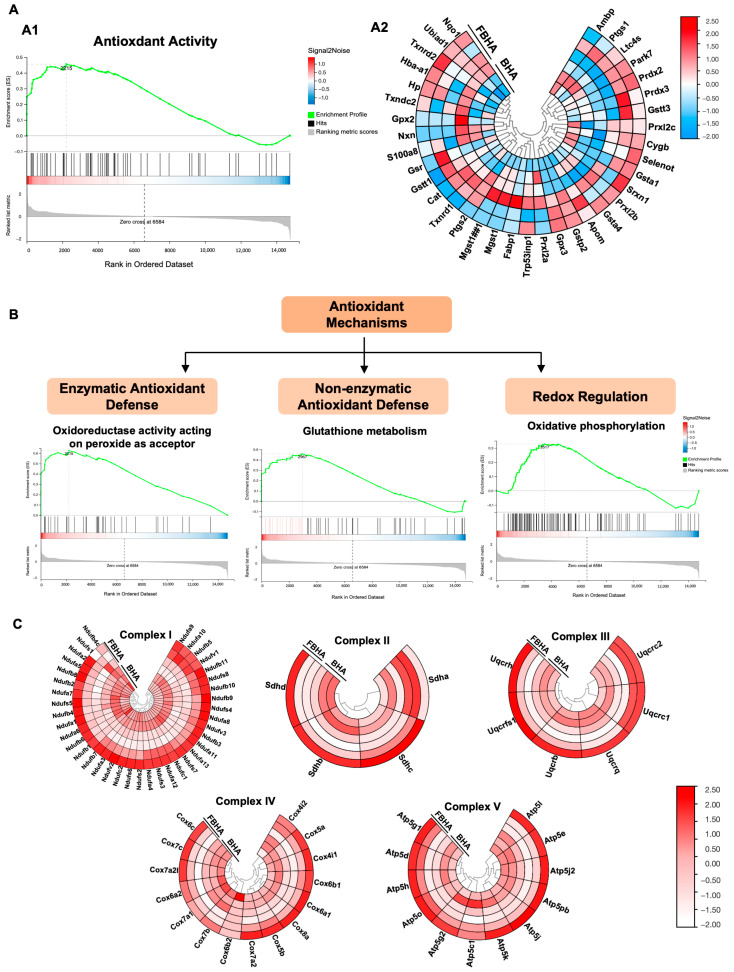
RNAseq analysis revealed the potential mechanisms by which FBHA inhibits the M1 polarization of macrophages. (**A**) GSEA (**A1**) and Associated Clustering Heatmap (**A2**) showed that, compared to PHA, the pathway of antioxidant activity of FBHA was significantly promoted. (**B**) The antioxidant mechanism involved several pathways, including oxidoreductase activity, glutathione metabolism, and oxidative phosphorylation (**C**), which might involve the enhancement of complex I, II, III, IV, and V in the mitochondrial respiratory chain of cellular energy metabolism.

RNA sequencing analysis was employed to elucidate the potential mechanisms through which FBHA inhibits the early-stage (6 h) M1 polarization of macrophages. Through integrated analysis employing GSEA and hierarchical clustering visualization, we identified that, compared to BHA, FBHA significantly enhanced the antioxidant activity pathway (Figure 3A), involving several pathways (Figure 3B), including oxidoreductase activity, glutathione metabolism, and oxidative phosphorylation. Furthermore, multiple mitochondrial respiratory chain complexes were upregulated (Figure 3C), including Complex I (NADH dehydrogenase), Complex II (Succinate Dehydrogenase), Complex III (Cytochrome C oxidoreductase), Complex IV (Cytochrome C oxidase), and Complex V (ATP synthase). It is reported that the polarization phenotypes and inflammatory responses of macrophages are closely associated with the cellular antioxidant activity, which in turn are regulated by the oxidoreductase activity of the cellular respiratory chain and the process of energy metabolism [22,23]. Consequently, drawing from these aforementioned findings, we hypothesize that FBHA modulated the oxidoreductase activity of macrophage mitochondria, influencing respiratory chain function and cellular energy metabolism, which resulted in heightened antioxidant activity, diminished oxidative stress and inflammatory responses, and ultimately regulated the polarization shifts in macrophages.

### 3.4. FBHA Promoted Osteogenic Differentiation via Modulating Macrophage Polarization

**Figure 4:**
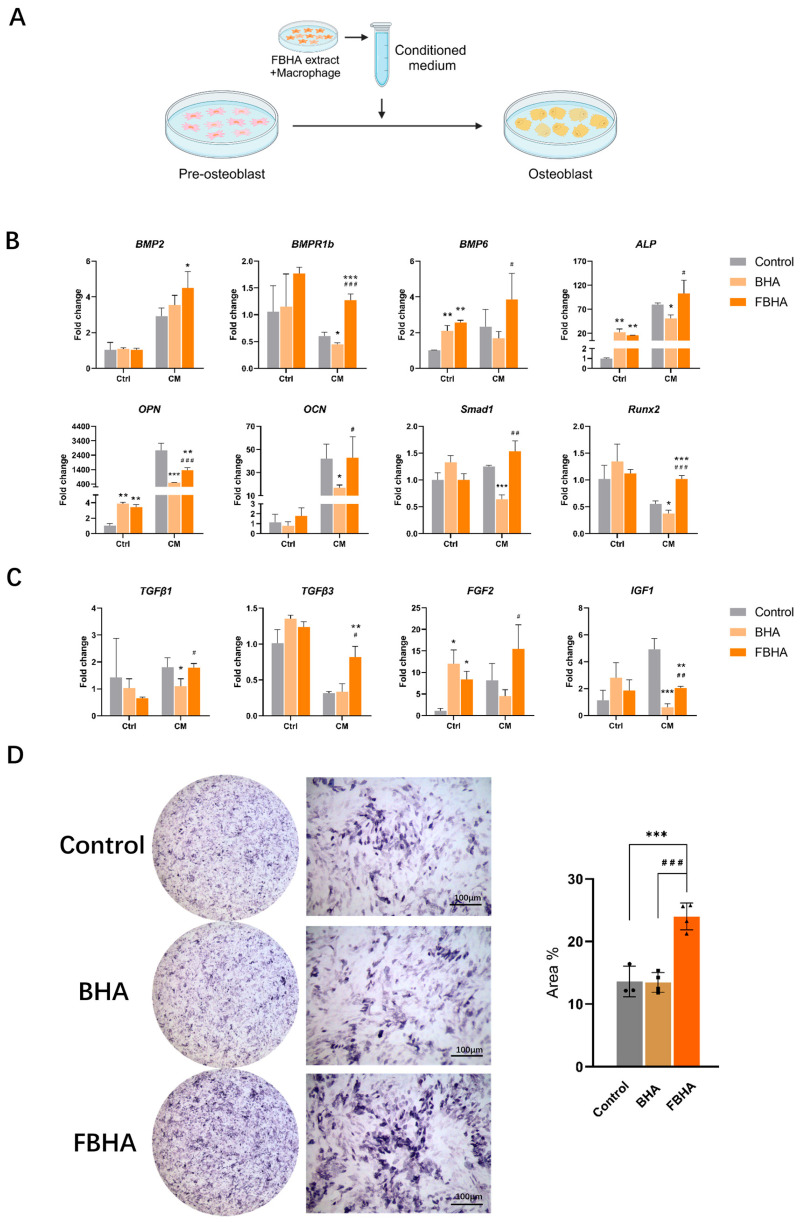
Osteogenic differentiation of pre-osteoblasts in FBHA and BHA. (**A**) In vitro model of material extract culturing with macrophages; RT-aPCR showed that (**B**) FBHA group upregulated the osteogenesis-related genes expression, and (**C**) osteogenesis-related growth factor gene expression. (**D**) ALP staining showed a significant enhancement of osteogenic differentiation in the FBHA group. *p* value: Compared with the Control group, *, *p* < 0.05; **, *p* < 0.01; ***, *p* < 0.001. Compared with BHA group, #, *p* < 0.05; ##, *p* < 0.01; ###, *p* < 0.001.

First, the RT-qPCR results revealed significant differences in the impact of FBHA and BHA on the osteogenic differentiation of pre-osteoblasts with the presence or absence of M1-polarized macrophages, which demonstrated that macrophages play a crucial role in BHA-related osteogenesis progress. Second, within the presence of macrophage-conditioned medium, compared to the Control group, the gene expression levels of osteogenic differentiation were significantly downregulated in the BHA group, including *BMPR1b*, *ALP*, *OPN*, *Runx2*, *Smad1*, *IGF1*, and *TGFβ1* (Figure 4B,C). In contrast, compared to the Control group, FBHA distinctly upregulated the gene expression of *BMP2*, *BMPR1b*, *Runx2*, *TGFβ3,* and *FGF2*, while concurrently downregulating *OPN* and *IGF1*.

Furthermore, it is interesting to note that, within the presence of macrophage-conditioned medium, compared to BHA, FBHA significantly upregulated the gene expression level of osteogenic markers *BMPR1b*, *BMP6*, *ALP*, *OPN*, and *OCN*, the osteogenic transcription factor *Smad1* and *Runx2* (Figure 4B), as well as growth factors *TGFβ1*, *TGFβ3*, *FGF2,* and *IGF1* (Figure 4C). Subsequently, after a 7-day co-culture period, ALP staining analysis demonstrated that FBHA significantly enhanced ALP activity compared with BHA (Figure 4D), which consisted of the gene expression of *ALP* with macrophage-conditioned medium. It has been reported that BMP6 might activate the Smad1/Runx2 signaling pathway via BMPR1B, thereby promoting osteogenesis-related gene expression such as *ALP*, *OPN*, and *OCN* [24]. In addition, TGFβ1/3 [25], FGF2 [26], and IGF1 [27] also play important roles in the regulation of MSCs’ osteogenic differentiation. These results illustrated that FBHA facilitated osteogenic differentiation of pre-osteoblasts via macrophage modulation.

Additionally, in contrast to the co-culture model that includes macrophage-conditioned medium, in the control model where material extracts were directly cultured with pre-osteoblasts, both FBHA and BHA significantly upregulated the gene expression of *BMP6*, *ALP*, *OPN*, and *FGF2*, with no significant effect on *BMP2*, *BMPR1b*, *OCN*, *Runx2*, *Smad1*, *TGFβ1*, and *IGF1*. Moreover, the gene expression levels between FBHA and BHA showed no significant differences. These findings suggested that studies only focusing on the direct effects of biomaterials and osteoblasts have overlooked the pivotal role of macrophages. Further, establishing a co-culture model that incorporates macrophages between biomaterials and osteoblast-related cells could be a more effective strategy to accurately simulate the early osteogenic microenvironment after biomaterial implantation and elucidate the specific interactions between the host and biomaterials.

In summary, within an M1 macrophage-dominated microenvironment, compared to BHA, FBHA effectively enhanced the osteogenic differentiation of pre-osteoblasts.

### 3.5. FBHA Enhanced Bone Regeneration and Biomineralization in Alveolar Sockets

#### 3.5.1. Radiological Evaluation

**Figure 5:**
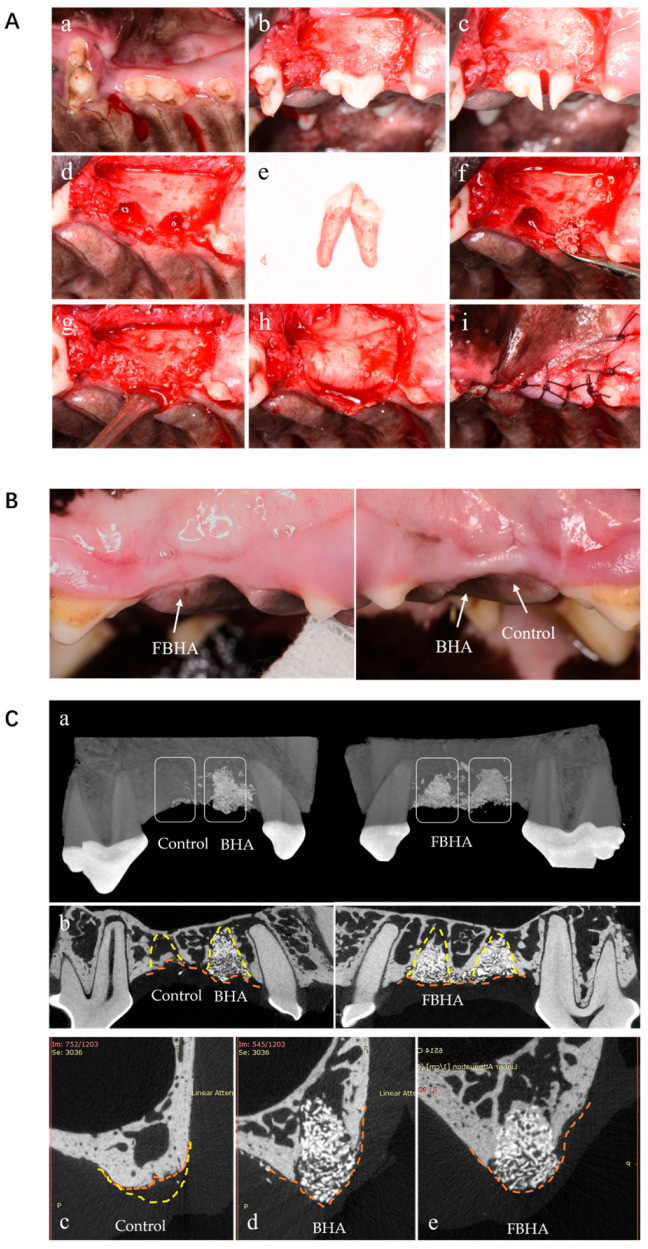
Socket preservation model in canine. (**A**) The establishment of an experimental animal model and surgery procedures, preoperative (**a**), flap reflection (**b**), tooth sectioning and extraction (**c**–**e**), bone grafting (**f**,**g**), collagen membrane placement (**h**), Suture (**i**), (**B**) The observation of the alveolar ridge contour and gingival healing status, (**C**) 3D reconstruction (**a**), sagittal (**b**) and coronal (**c**–**e**) plane observation of micro-CT showed collapsed contours in the Control group (**c**) and well-maintained alveolar contours in the BHA (**d**) and FBHA (**e**) groups. Yellow dashed line: Alveolar socket margin in (**C**(**b**)) and preconceived ridge contour in (**C**(**c**)); Orange dashed line: Alveolar ridge contour in (**C**(**b**–**e**)).

As clinical observation showed, no signs of inflammation, infection, or wound dehiscence were observed during the entire experiment. After 3 months of uneventful healing, the edentulous ridges were covered with a tough and resilient mucosa as normal (Figure 5B). The alveolar ridge profile and texture at the grafted sites seemed superior to the non-grafted sites, whereas no obvious difference was observed between FBHA and BHA sites. In 3D visualization micro-CT evaluation, the contours of alveolar ridges in the FBHA and BHA groups were better preserved, whereas the ridge atrophy was obvious in the Control group (Figure 5C). The bone substitute particles mainly remained in sockets, and NB could be observed around graft particles inside the sockets.

**Figure 6:**
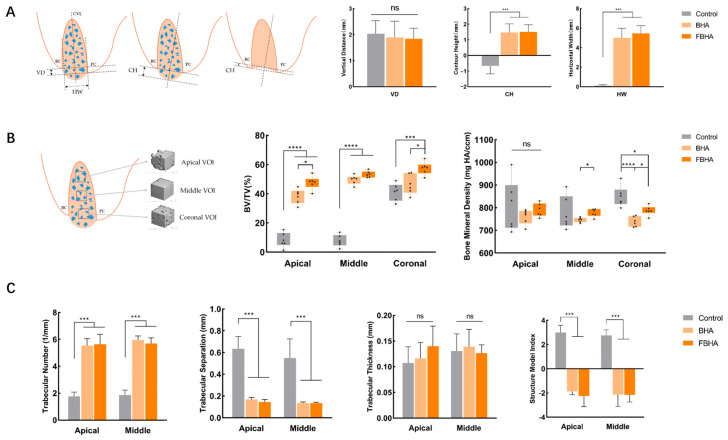
Micro-CT measurement analysis. (**A**) the alveolar bone contour measurement showed a well-restored bone contour in both the FBHA and BHA groups, (**B**) ROI analysis inside the sockets demonstrated that higher percentage of new bone formation and higher degree of bone mineralization were observed in the FBHA group, (**C**) Trabecular analysis indicated no significant difference between the BHA and FBHA groups. *p* value: *, *p* < 0.05; ***, *p* < 0.001, ****, *p* < 0.0001; ns, no significant difference.

In terms of alveolar ridge contours, the VD, CH, and HW were shown (Figure 6A). There was no significant difference in VD among the three groups, which revealed that bone resorption of the original alveolar buccal crest could not be avoided by biomaterials’ grafting. However, the CH and HW of the grafted groups were significantly higher than the Control group, while no difference was observed between FBHA and BHA, suggesting that the alveolar ridge contour was successfully maintained because of the space maintenance capacity of FBHA and BHA particles with a similar low degradation rate. On the contrary, collapsed and atrophic alveolar ridge contours were also observed in the Control group, demonstrating the necessity of post-extraction socket preservation.

In addition, we also focused on bone healing within the extraction socket. The BV/TV and BMD of all VOIs were shown as follows (Figure 6B). The value of BV/TV represented the quantity of new bone formation. Compared to the Control group, the BV/TV of the FBHA group was significantly greater in the apical, middle, and coronal VOIs, whereas in the BV/TV of the BHA group, they were significantly greater only in the apical and middle VOIs. Interestingly, it was found that the BV/TV of the FBHA group was significantly greater than the BHA group in both the apical and coronal VOIs. In the middle VOI, there were no significant differences between the FBHA and BHA groups, although the average of the FBHA group was slightly higher. These results indicated that FBHA promoted new bone formation in the sockets. Furthermore, in the Control group, the BV/TV in the apical and middle VOIs were obviously lower than the coronal VOI, in accordance with the cortical bone bridge formed at the entrance of the socket rather than on the sparse trabeculae inside the socket, which reflected the general healing state of non-grafted sockets. Meanwhile, the values of BV/TV gradually increased from the apical to coronal VOIs in all groups, indicating the different healing patterns in different regions of sockets.

The value of BMD represented the quality of new bone formation, as well as bone maturation. In the apical, middle, and coronal VOIs, the BMD of the Control group seemed to be the highest among the three groups, consistent with the consensus that HA-related grafts delay the bone healing process of the extraction socket. Remarkably, the BMD of the FBHA group was significantly higher than the BHA group in the middle and coronal VOIs, although the average of the FBHA group was slightly higher than the BHA group in the apical VOI.

**Figure 7:**
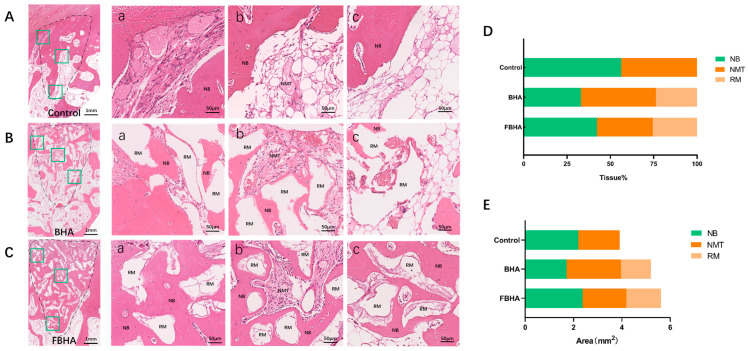
Histomorphological evaluation. (**A**–**C**) H&E staining showed greater new bone formation and more mature bone microstructure in FBHA compared with BHA. (**D**,**E**) Histomorphological measurement demonstrated a higher rate of new bone percentage (**D**) and area (**E**) in FBHA. Dashed line: extraction socket area; Green box: coronal (**a**), middle (**b**) and apical (**c**) regions in the socket. NB: new bone; RM: residue material; NMT: non-mineral tissue.

As regards the healing pattern of different areas inside the socket, the values of BMD implied the disparity of bone maturation. For the Control group, the bone mineral density appeared to be the highest in the coronal VOI, followed by the apical VOI, with the middle VOI showing the lowest values. This suggested that during the natural healing process, the cortical bone formed in the coronal area possesses a more mature mineralized structure, which can be interpreted as the dual promotional effect on bone healing from the periosteum and the socket wall’s blood supply and stem cells in the absence of a xenograft implant. Whereas the center of the middle region of the socket, being farther from the socket bone wall, exhibited relatively slower bone maturation, which was also confirmed in the subsequent histological sections (Figure 7). In addition, there were differences between the grafted and non-grafted groups. For the FBHA and BHA groups, the BMD in the apical VOI appeared to be the highest, with the middle VOI being similar to the coronal VOI, which not only illustrated the interference of grafts implantation on the general bone maturation of extraction socket, but also revealed that FBHA could improve BHA-related deficiencies in biomineralization.

Concerning trabecular bone morphometry inside the sockets, significant differences were observed between the grafted and non-grafted group in Tb.N, Tb.Sp, and SMI, except for Tb.Th (Figure 6C). No significant differences were found between the FBHA and BHA groups in both apical and middle VOIs, indicating that the grafted groups showed similar trabecular bone space and structure. The Tb.N, Tb.Sp, and SMI of the FBHA and BHA groups were significantly different from the Control group. Specifically, the thickness of the trabeculae was similar in the FBHA, BHA, and Control groups, whereas the presence of the grafts led to an increase in trabecular number and a decrease in trabecular spacing. Meanwhile, the results of the SMI suggested that the trabecular bone structure in the FBHA and BHA groups tended to be plate-like, and the trabeculae of the Control group tended to be rod-like. These results of trabecular bone morphometry analysis demonstrated that the FBHA and BHA groups showed intensively arranged trabeculae, while the Control group exhibited sparse trabeculae with wide marrow space.

#### 3.5.2. Histological Observation and Histomorphometric Analysis

In histological sections, it was noted that FBHA and BHA particles implanted into the extraction sites were stably retained within the sockets, surrounded by either newly formed bone tissue or non-mineralized tissue (Figure 7). Additionally, the dimensions of the particles for both FBHA and BHA were found to be similar (Figure 7B,C).

Histomorphometric analysis revealed that, when assessed statistically by percentage (Figure 7D), the FBHA group exhibited a higher percentage of new bone formation than the BHA group, while the Control group showed the highest percentage of new bone due to the absence of bone grafts. However, when measured by actual area size (Figure 7E), the new bone area of the BHA group was found to be lower than the Control group, aligning with previous research indicating that HA implantation can delay the bone healing process within the extraction socket. Conversely, the new bone area of the FBHA group was not only higher than the BHA group but also comparable to the Control group. Both percentage and area measurement analyses demonstrated the enhanced bone regeneration of FBHA. Additionally, the measurement of actual area also indicated that the Control group without bone grafts experienced atrophy of the alveolar socket area. This suggested that the use of bone grafts could be of benefit for maintaining the alveolar ridge contour, which was consistent with the radiological results above.

Furthermore, upon high magnification examination, we observed the different patterns of bone healing and maturation among the coronal, middle, and apical regions of the sockets. In the coronal region, the Control group displayed a more substantial amount of newly formed cortical bone in the entrance of the socket, covered by a periosteum-like tissue, with the concurrent infiltration of non-ossified fibrous connective tissue (Figure 7A(a)). Both FBHA and BHA particles were surrounded by new bone, and osteoclast-like multinucleated giant cells were observed at the surface of particles without bone tissue surrounds (Figure 7B(a),C(a)), indicating the low degradation rate of FBHA and BHA. Moreover, in comparison to BHA, FBHA demonstrated a more extensive area of new bone formation and presented a more regular structure with a Haversian system, with bone depositing around the particles layer by layer, suggesting that FBHA facilitated new bone deposition and mineralization, which corroborated the results of the micro-CT evaluation.

In the middle region of the socket, a central area occupied by non-mineralized tissue and rich in blood vessels was noted in all groups, surrounded by newly formed bone tissue (Figure 7A(b),B(b),C(b)). Notably, the NMT area in the FBHA group was markedly reduced compared to the BHA group. On the one hand, this can be interpreted as the bone healing of the extraction socket starting from the socket wall toward the center to achieve complete bone healing. On the other hand, it also indicated that fluoridation improved the bone healing efficiency of BHA inside extraction sockets.

In the apical region, in addition to bone tissue and implanted biomaterials, a small amount of marrow-like tissue was observed in the FBHA group (Figure 7C(c)). In contrast, the BHA and Control groups exhibited an abundance of adipose-like marrow tissue along with a few new bone tissues (Figure 7A(c),B(c)). Similarly, a large amount of marrow tissue was noted in the middle region of the Control group.

Overall, the socket of the BHA group was largely occupied by non-mineralized tissue and material particles, with a certain amount of newly formed bone. In contrast, the socket healing pattern of the FBHA group was predominantly characterized by the formation of newly formed mineralized bone, which confirmed the superiority of fluoride doping as a modification strategy for BHA in promoting bone healing and maturation.

## 4. Discussion

The socket preservation technique using biogenic hydroxyapatite (BHA)-based biomaterials has been widely applied in clinical practice. However, the early pro-inflammatory properties and the delayed bone healing process associated with BHA have not yet been addressed [3,7]. This study aimed to improve the aforementioned shortcomings of BHA by fluorine incorporation, and to the best of our knowledge, it is the first time that fluoridation has been verified to indeed effectively strengthened bone healing of BHA in extraction socket preservation in canine by improving the macrophage-mediated immune response.

The canine socket preservation model is widely regarded as an appropriate and classic model for simulating human tooth extraction healing, as both share similarities in socket morphology and healing patterns [1]. Meanwhile, differences exist in the healing speed and tissue remodeling dynamics between dogs and humans. Notably, it has been reported that the remodeling of periodontal ligament fibers in dogs occurs more rapidly, and 8-week socket healing in dogs corresponds to a similar degree of bone remodeling, and healing is 24 weeks or longer in humans [3]. Additionally, dogs exhibit a propensity for significant buccal bone plate resorption, mirroring the clinical scenario in humans where the thinner buccal bone plate in the anterior region often leads to more pronounced vertical and horizontal bone loss [2]. Consequently, the canine socket preservation model serves as a reliable preclinical animal study model for the performance evaluation of dental biomaterials.

To date, one of the most common clinical strategies to address delayed healing in extraction sockets involves the use of collagen-based biomaterials as an alternative to biogenic hydroxyapatite [28], which is particularly effective in specific situations, such as posterior tooth regions with thick buccal bone. However, due to the deficiency of an osteogenic space maintenance ability in the collagen scaffold, BHA remains the preferred choice in most cases. Therefore, mitigating the early inflammatory response of BHA has emerged as a critical research priority.

Several strategies have been reported to improve the osteoimmune response of biogenic hydroxyapatite, including ion doping [29], modifying sintering temperature [7], controlling shape and size [30], and incorporating hydrogel-based additives [31]. Since the human skeletal system naturally contains trace elements [32], it is reported that ion doping, such as fluorine (F) [15], magnesium (Mg) [33], and zinc (Zn) [34], not only alleviates inflammation but also introduces additional biological benefits [29]. Among these, fluoride doping has been demonstrated to enhance the physicochemical and biological properties of BHA, making it a promising modification strategy [16,17,18].

In this work, fluoride ions were successfully doped into BHA through chemical immersion and high-temperature sintering to obtain FBHA. Regarding the mechanism of fluoride ion doping in hydroxyapatite, our earlier studies confirmed that fluorine incorporation occurred through the substitution of hydroxyl groups within the apatite lattice via utilizing XRD and FTIR analyses [14,17,18]. Specifically, the increased fluoridation correlates with enhanced crystallinity and a gradual reduction in the a-axis lattice constant, while the c-axis lattice constant remains unchanged. These structural modifications derive a morphological transition from spherical to rod-like crystals, enabling stable lattice doping rather than superficial fluoride adsorption [17]. This phenomenon of lattice contraction in FBHA arose from the partial substitution of hydroxyl groups by F^−^, primarily attributed to the smaller ionic radius of fluoride ions, stronger ionic bonding, and structural densification effects.

Moreover, the fluoridation of BHA resulted in a sustained and slow release of fluoride ions. Further optimization of fluoride concentration yielded fluoridated BHA with superior compressive strength, reduced solubility, and sustained fluoride ion release capacity, which is considered to be advantageous for actively modulating osteogenic microenvironments around materials [14,16,18]. Building on this groundwork, in this study, we prepared FBHA using the previously validated optimal fluoride concentration, and the results of physicochemical characterization in this study were also consistent with the previous work, confirming the successful incorporation of fluoride ions. We noted the detection of carbon in the results of EDS, consistent with our previous research [16,17,18]. It has been reported that carbon in biogenic hydroxyapatite is present as carbonate ions [35].

FBHA and BHA extracts were used in the in vitro study. Regarding the ion release in extracts, it has been reported that [17], despite its reduced solubility, FBHA crystals ultimately reached a dynamic equilibrium between dissolution and recrystallization in solution, during which multiple ions were released, including fluoride, magnesium, calcium, and phosphate ions. Regarding the ion release of fluorinated BHA, our previous studies have confirmed that FBHA and BHA extracts exhibited reduced release of calcium and phosphate ions but increased magnesium ion release [16,18]. In fact, the adsorption of calcium and phosphate ions from solution onto biogenic HA material surfaces is a well-documented physicochemical phenomenon [36]. Meanwhile, compared to BHA, the FBHA extract contained the unique presence of fluoride ions, along with significantly higher concentrations of magnesium and calcium ions.

On the whole, we found that FBHA significantly boosted the bone healing process of extraction socket sites via addressing the BHA-related inflammation response. Specifically, FBHA demonstrated superior performance in promoting bone regeneration and bone mineralization, which involved the immunomodulation of the early osteogenic microenvironment by regulating macrophage polarization. By mitigating the early inflammatory response associated with BHA, FBHA effectively balanced immune reactions and bone healing processes, ultimately facilitating bone repair.

Mechanistically, the results of the in vitro model revealed that FBHA might exert its effects by releasing trace fluoride ions that regulate macrophage polarization within the osteogenic microenvironment. In particular, FBHA appeared to correct the BHA-related early inflammatory response by directly suppressing the level of the pro-inflammatory response associated with M1 macrophage, which might facilitate a shift from M1 toward an M2-dominant phenotype. Subsequently, through macrophage-associated inflammatory regulation, BHA inhibited the osteogenic differentiation of pre-osteoblasts by downregulating the expression of key osteogenic genes, including *BMPR1b*, *ALP*, *OPN*, *Smad1*, *Runx2*, *TGFβ1*, and *IGF1*. In contrast, FBHA addressed the M1-related inflammatory response, then enhanced the osteogenic differentiation of pre-osteoblasts. These results were consistent with our previous finding that fluoride ions can act as osteoimmunomodulatory agents to promote osteogenesis [15]. Additionally, in this study, the fluoride ion release concentrations of FBHA were precisely close to the range of 2.4 to 24 μM, reported by Wu’s research [15] as the optimal concentration for the regulation of osteoimmune microenvironment, which reflected the biological efficacy of the in situ ion release of FBHA in the early stage of macrophage-mediated osteoimmunomodulation. Studies have also shown that the in situ ion release from apatite biomaterials is conducive to driving the in situ bone regeneration [18,37].

However, although the effect of fluoride ions alone on osteogenesis in the rat calvarial defect model has been demonstrated in our previous study, the osteogenic efficacy of fluoride-doped BHA in larger animal models remained unclear. It is widely acknowledged that small animal models frequently serve for the preliminary assessment of biomaterials [38], whereas large animal models hold immense significance for the preclinical investigations of biomaterials [39]. To address this gap, the effect of FBHA was evaluated in a canine jawbone socket preservation model for the first time. Remarkably, FBHA boosted the new bone formation and biomineralization, which highlighted its advantages in promoting bone healing of extraction sockets. As far as we know, fluoride-modified BHA also achieved better ridge contour maintenance and bone regeneration in lateral ridge augmentation [40] and peri-implant bone defects in canine [41]. Collectively, fluorine doping enhanced alveolar socket bone healing through addressing the macrophage-mediated inflammatory-immune response caused by biogenic hydroxyapatite.

This study is the first to validate the bone regenerative potential of FBHA in a canine model, providing experimental evidence for its potential clinical application in humans. As an optimized alternative to conventional BHA, FBHA demonstrates significant clinical value in both standalone and adjunctive bone regeneration therapies. Recent randomized controlled trials indicate that combining platelet-rich fibrin with demineralized freeze-dried bone allograft (DFDBA) does not significantly enhance clinical outcomes over DFDBA alone [42]. As a bioactive and osteoconductive material, FBHA offers structural support and ionic signaling to modulate the regenerative microenvironment, suggesting its potential to synergize with bioactive factors for improved bone regeneration. In the context of complex bone defect repair, such as alveolar reconstruction in cleft lip and palate patients, autologous bone grafting derived from the iliac bone or chin bone remains the gold standard. However, its use is limited by donor site morbidity and graft resorption [43]. With the bioactivity and strong bone regeneration potential, FBHA emerges as a promising alternative.

Additionally, with the rapid advancement in veterinary dentistry, the demand for precise bone grafting solutions in animals is increasingly recognized, shifting from life-saving to a new era emphasizing both functionality and aesthetics. Bone grafting in small companion animals (e.g., small dogs, cats) can significantly reduce post-extraction bone resorption, preserve the alveolar ridge structure, and provide a foundation for subsequent restorations (e.g., dental implants), thus reducing the risk of mandibular fractures. Given their thinner alveolar bone and weaker healing capacity, it is particularly critical that the in situ release of bioactive ions from hydroxyapatite, such as fluoride ions, can promote bone defect regeneration and enhance biomineralization, thereby effectively reducing the healing period and complications. The findings of this study provide valuable insights into the clinical application of FBHA in this field. Other studies have demonstrated that hydroxyapatite–glycosaminoglycan composites effectively promote bone regeneration and reduce complications in canine bone defects following tooth extraction [44]. This evidence lays the foundation for further exploration of FBHA-based composite strategies incorporating moldable carriers to enhance both biofunctionality and handling properties in future clinical applications.

Macrophage is considered to play an important role in the immune regulation of the early bone regeneration process [7,10,30]. Thus, it is essential to incorporate macrophages into the in vitro assessments of the osteoimmune performance of biomaterials. The balance of macrophage polarization is critical for local bone regeneration and the prognosis of biomaterials [11]. In other words, reducing M1 polarization and promoting M2 polarization in the early stage might benefit bone healing. In this study, the M1-dominated macrophage in vitro model was obtained successfully by using LPS activation to simulate the microenvironment of postoperative wounds [19]. The results revealed that the presence or absence of macrophages significantly influenced the outcomes of the in vitro study, underscoring the importance of including macrophages in the in vitro evaluation model.

In an in vitro experiment, M0 macrophages co-cultured with BHA extract exhibited upregulated pro-inflammatory gene expression (*IL6*, *TNFα*) and downregulated *Arg1* compared to the Control group, aligning with the BHA’s pro-inflammatory effects. However, in the LPS-stimulated M1 model, BHA extract downregulated not only pro-inflammatory markers (*iNOS*, *IL6*, *TNFα*) but also anti-inflammatory genes (*Arg1*, *IL10*, *TGFβ1*), totally different from M0 response patterns. Based on our current knowledge, this divergence might be attributed to the factors below.

First, in the LPS-stimulated M1 model, pro-inflammatory gene expression levels of the Control group were significantly upregulated (*iNOS* 10^3^-fold, *IL6* 10^6^-fold, *TNFα* 10^2^-fold) (Figure 2C), compared with the Control group in the M0 model, which indicated the intense baseline inflammation in M1 macrophages triggered by LPS. Interestingly, the concurrent moderate upregulation of anti-inflammatory genes (*IL10* 32-fold, *TGFβ* 7-fold) (Figure 2D) in the Control group potentially reflected immune homeostasis mechanisms [45] in which excessive inflammation could trigger compensatory IL10/TGFβ1 secretion via STAT3/NF-κB regulation [46]. Second, material extracts were used in the in vitro experiment. It is reported that BHA’s pro-inflammatory effects are mainly attributed to the characteristics of material surfaces, such as surface microstructure [47] and calcium-rich surfaces [7], which generally requires the materials’ direct contact with cells. However, in the absence of direct material-cell contact, macrophage immune response can also be regulated by ions in the extract. Our previous work [16,18] revealed that BHA extracts exhibited significantly reduced calcium ions, and slightly increased magnesium ions, which showed potential correlation with the trends of pro/anti-inflammatory gene expression in M1 macrophages. Calcium ion concentration has been well-characterized as a critical regulator of macrophage-mediated inflammation by activating TLR4-mediated inflammatory pathway through promoting extracellular calcium influx [7]. Meanwhile, the reduction in extracellular calcium ion concentration will lead to diminished calcium influx, which mediates the attenuation of inflammation, suggesting that the decreased calcium level in the BHA extract might potentially inhibit inflammatory responses. In addition, although magnesium ions possess anti-inflammatory properties, the cumulative magnesium ions release in BHA extract was not significant [16]. Therefore, the reduction in calcium ion concentration appears to be the critical factor mediating the downregulation of pro-inflammatory gene expression (*iNOS*, *IL6*, *TNFα*) in BHA extract-treated M1 macrophages. After that, the concurrent downregulation of anti-inflammatory gene expression (*Arg1*, *IL10*, *TGFβ*) might be associated with macrophage recovery from hyperinflammatory states.

Notably, compared to the baseline of BHA, the FBHA extract further suppressed M1 inflammation markers (*iNOS*/*TNFα* downregulation), NO production, and phagocytic activity. Critically, fluoride doping enhanced the immunomodulatory capacity of the FBHA extract by an increased release of fluoride and magnesium ions [16,18]. These are aligned with fluoride ions’ documented role in macrophage polarization modulation [15]. Furthermore, the alveolar ridge preservation experiment in the canine model demonstrated FBHA’s superior bone regeneration outcomes, with elevated values in the BV/TV and BMD of micro-CT analyses, which was potentially mediated through fluoride doping’s dual function: mitigating macrophage-driven inflammation and promoting osteogenic mineralization. In terms of the mechanisms underlying FBHA-mediated macrophage modulation, the result of early RNA-seq analysis indicated that FBHA enhanced the oxidoreductase activity and antioxidant activity of macrophages, as well as multiple complexes of the mitochondrial respiratory chain. It is well-documented that macrophage polarization and the inflammatory response of macrophages are regulated by the cellular antioxidant activity [22,48]. Therefore, we hypothesize that the ionic components in FBHA extract substantially affect the mitochondrial respiration and antioxidant activity of M1 macrophages, thereby alleviating M1-related NO release and inflammatory responses. The specific pathways involved still remain to be further investigated in future experiments.

iNOS is reported to be responsible for producing NO during inflammatory responses, which plays a crucial role in immune inflammation [49]. In this study, different trends in *iNOS* gene and NO expression were observed between the FBHA and Control groups, which might stem from the fact that NO homeostasis is regulated through multiple pathways with high redundancy. These pathways might include the enhanced catalytic efficiency of iNOS associated with cofactors tetrahydrobiopterin [50], the compensation from other nitric oxide synthases [51], and the increased superoxide dismutase activity [52]. Given that the transcriptional regulation of individual genes alone cannot comprehensively characterize NO biosynthesis dynamics, this study simultaneously measured both *iNOS* gene expression and NO release level to comprehensively and objectively evaluate the immunoinflammatory effects of FBHA and BHA.

These are also limitations in this study. First, extracts of biomaterial were used for co-culturing with cells instead of biomaterial particles directly with cells. Culturing cells directly with material particles is challenging. Some studies explored co-culturing models with a small amount of processed material particles or with microfluidics to create a stable platform [7,53]. However, these models still have similar drawbacks, such as alterations in the quantity and surface morphology of biomaterials. Therefore, extracts were adopted in the in vitro models to evaluate the effect of trace ion release by apatite-based FBHA and BHA on macrophage-mediated biogenesis. In terms of the component of material extract, except F ions that played a primary physiological role, FBHA extracts also demonstrated alterations in the increased release of Mg ions, as our previous study showed [16], which might collectively contribute to the pro-osteogenesis property of FBHA. In addition, we found that the fluoride ion release of FBHA exhibited an initial high concentration in the early stage, which gradually decreased to a more stable concentration over time. Therefore, the 24 h FBHA extract with the highest concentration was used as the subject in this experiment. However, under the conditions of body fluid circulation, the dissolution equilibrium and ion release of the biomaterials may differ and change over time [54]. Yet, the extracts in this study cannot fully simulate the in vivo microenvironment, which remains an inevitable limitation of in vitro research. Additionally, the regulatory mechanisms of macrophage polarization were discussed. The hypotheses presented in this paper might be related to cellular respiration and mitochondrial function, yet these remain to be further explored and validated in future research.

## 5. Conclusions

In this study, compared to BHA, FBHA significantly inhibited the inflammatory response of M1 macrophages, which promoted the osteogenic differentiation of pre-osteoblasts via addressing the BHA-associated pro-inflammatory microenvironment, ultimately facilitating socket bone healing in a canine model. This study offered a deeper understanding of the role of fluorine doping into biogenic hydroxyapatite on osteoimmunomodulation and bone regeneration. The findings exhibited important implications for the advancement of fluoride-modified biogenic hydroxyapatite, providing insights into the development of next-generation biomaterials for socket preservation and potential clinical applications.

## Data Availability

The data presented in this study are available upon reasonable request from the corresponding author.

## Supplementary Materials

The following supporting information can be downloaded at: https://www.mdpi.com/article/10.3390/bioengineering12040396/s1, Table S1. Primer sequences of RAW264.7; Table S2. Primer sequences of MC3T3-E1.
