# Supplementary material for: Fluorine-Incorporated Biogenic Hydroxyapatite Enhances Socket Bone Healing via Addressing Macrophage-Mediated Inflammatory Response
Source: Bioengineering (Basel). 2025 Apr 7;12(4):396. doi: 10.3390/bioengineering12040396 (PMC12024827; doi:10.3390/bioengineering12040396)
Supplement: Supplementary file 1 [file bioengineering-12-00396-s001.zip › bioengineering-3498071-supplementary.pdf]

Table S1. Primer sequences of RAW264.7.

Gene	Forward	Reverse
iNOS	CAGAAGTGCAAAGTCTCAGACAT	GTCATCTTGTATTGTTGGGCT
IL6	ATAGTCCTTCCTACCCCAATTTCC	GATGAATTGGATGGTCTTGGTCC
TNFα	CTGAACTTCGGGGTGATCGG	GGCTTGTCACTCGAATTTTGAGA
Arg1	ATCAACACTCCCCTGACAAC	GCCAATGTACACGATGTCTTTG
IL10	GAGAAGCATGGCCCAGAAATC	GAGAAATCGATGACAGCGCC
TGFβ1	GTGGAAATCAACGGGATCAGC	CAGCAGTTCTTCTCTGTGGAGC
β-actin	CATACCCAAGAAGGAAGGCTGG	GCTATGTTGCTCTAGACTTCGAGC

Table S2. Primer sequences of MC3T3-E1.

Gene	Forward	Reverse
BMP2	CGGACTGCGGTCTCCTAA	GGGGAAGCAGCAACACTAGA
BMPR1b	GACACTCCCATTCCTCATC	GCTATTGTCCTTTGGACCAG
BMP6	TGTGAACCTGGTGGAGTACG	GGTGTCACCACCCACAGATT
ALP	GTTGCCAAGCTGGGAAGAACAC	CCCACCCCGCTATTCCAAAC
OPN	ATCTCACCATTCGGATGAGTCT	TGTAGGGACGATTGGAGTGAAA
OCN	CTGACAAAGCCTTCATGTCCAA	GCGCCGGAGTCTGTTCACTA
Smad1	CTACTGGCGCAGTCTGTGAA	GGGGTGCTGGTAACATCCT
Runx2	AGCCTCTTCAGCGCAGTGAC	CTGGTGCTCGGATCCCAA
TGFβ1	ATGCTAAAGAGGTCACCCGC	GTATCAGTGGGGGTCAGCAG
TGFβ3	GGACTTCGGCCACATCAAGA	ATAGGGGACGTGGGTCATCA
FGF2	GGCTGCTGGCTTCTAAGTGT	GTCCCGTTTTGGATCCGAGT
IGF1	AAAGCAGCCCGCTCTATCC	CTTCTGAGTCTTGGGCATGTC
β-actin	CATACCCAAGAAGGAAGGCTGG	GCTATGTTGCTCTAGACTTCGAGC
